# Topographic organization across foveal visual areas in macaques

**DOI:** 10.3389/fnana.2024.1389067

**Published:** 2024-04-29

**Authors:** Hangqi Li, Danling Hu, Hisashi Tanigawa, Toru Takahata

**Affiliations:** ^1^Key Laboratory for Biomedical Engineering of Ministry of Education, College of Biomedical Engineering and Instrument Science, Zhejiang University, Hangzhou, China; ^2^Interdisciplinary Institute of Neuroscience and Technology, School of Medicine, Zhejiang University, Hangzhou, China; ^3^Department of Neurology of the Second Affiliated Hospital, School of Medicine, Zhejiang University, Hangzhou, Zhejiang, China

**Keywords:** foveal visual field, feedback projection, retrograde labeling, striate cortex, *Macaca mulatta*

## Abstract

**Introduction:**

While the fovea on the retina covers only a small region of the visual field, a significant portion of the visual cortex is dedicated to processing information from the fovea being a critical center for object recognition, motion control, and visually guided attention. Despite its importance, prior functional imaging studies in awake monkeys often focused on the parafoveal visual field, potentially leading to inaccuracies in understanding the brain structure underlying function.

**Methods:**

In this study, our aim is to unveil the neuronal connectivity and topography in the foveal visual cortex in comparison to the parafoveal visual cortex. Using four different types of retrograde tracers, we selectively injected them into the striate cortex (V1) or V4, encompassing the regions between the fovea and parafovea.

**Results:**

V1 and V4 exhibited intense mutual connectivity in the foveal visual field, in contrast to the parafoveal visual field, possibly due to the absence of V3 in the foveal visual field. While previous live brain imaging studies failed to reveal retinotopy in the foveal visual fields, our results indicate that the foveal visual fields have continuous topographic connectivity across V1 through V4, as well as the parafoveal visual fields. Although a simple extension of the retinotopic isoeccentricity maps from V1 to V4 has been suggested from previous fMRI studies, our study demonstrated that V3 and V4 possess gradually smaller topographic maps compared to V1 and V2. Feedback projections to foveal V1 primarily originate from the infragranular layers of foveal V2 and V4, while feedforward projections to foveal V4 arise from both supragranular and infragranular layers of foveal V1 and V2, consistent with previous findings in the parafoveal visual fields.

**Discussion:**

This study provides valuable insights into the connectivity of the foveal visual cortex, which was ambiguous in previous imaging studies.

## Introduction

In the primate retina, there is a tiny pit responsible for the most central visual field (< 1.5 degrees), known as the fovea. Human eyes make saccades onto the visual scene, guiding motion and directing attention, ensuring that visual objects of interest fall into the fovea. Consequently, foveal vision is at the center of conscious vision in daily life (Knapen et al., 2016; Guzhang et al., 2021; Intoy et al., 2021). Nevertheless, previous studies on processing such information were mostly conducted on the parafoveal visual field or the mixture of foveal and parafoveal visual fields in the brain (Bastos et al., 2015; Doostmohammadi et al., 2023), which may lead to inaccuracies in understanding brain structure and connectivity. This is because precise mapping of visuotopy in the foveal visual field is technically and ethically challenging (Blasdel and Campbell, 2001; Nauhaus et al., 2016; McGregor et al., 2018).

Firstly, the visual field of the fovea is very small, and determining eye position precisely in anesthetized animals is almost impossible; therefore, visual experiments in anesthetized subjects are excluded. To study the foveal visual field, monkeys need to be trained to gaze at a fixed point for an extended period without anesthesia, while tiny visual stimuli are presented within or in the vicinity of the focus. However, micro-saccades may yet affect measurements in awake monkeys (Shelchkova and Poletti, 2020).

Secondly, high spatial resolution is required when studying the topography of foveal representation in the visual cortex through functional imaging. While a few studies used functional magnetic resonance imaging (fMRI) in humans and monkeys, they did not focus on millimeter or submillimeter-scale modular structures (Schira et al., 2009; Kolster et al., 2014). Even with electrophysiology and optical imaging, it remains challenging to distinguish differences within 1.5 degrees of eccentricity (Pinon et al., 1998; Chaplin et al., 2013). Additionally, the foveal visual areas are located near the ear in macaques, making surgical access for imaging difficult. The surgical procedure required for long-term imaging may result in damage to the temporalis muscle, which is necessary to access foveal regions. This raises significant ethical concerns related to feeding behavior during the recovery period.

Assuming that neurons of the striate cortex (V1) are interconnected with neurons of higher visual cortices that share the same receptive fields, anatomical tracing studies were performed to determine the topography of higher visual cortices by injecting tracers into V2 or V4 (Gattass et al., 1997; Ungerleider et al., 2008). However, classical studies have conducted one injection of tracer per animal to investigate the connectivity of higher visual cortices, while interindividual variability is substantial in primates, limiting precise topographic information. Even when two or three types of tracers were used in a single animal, the tracers were different chemical reagents, which may behave differently *in vivo* (Stepniewska and Kaas, 1996).

**Figure 1:**
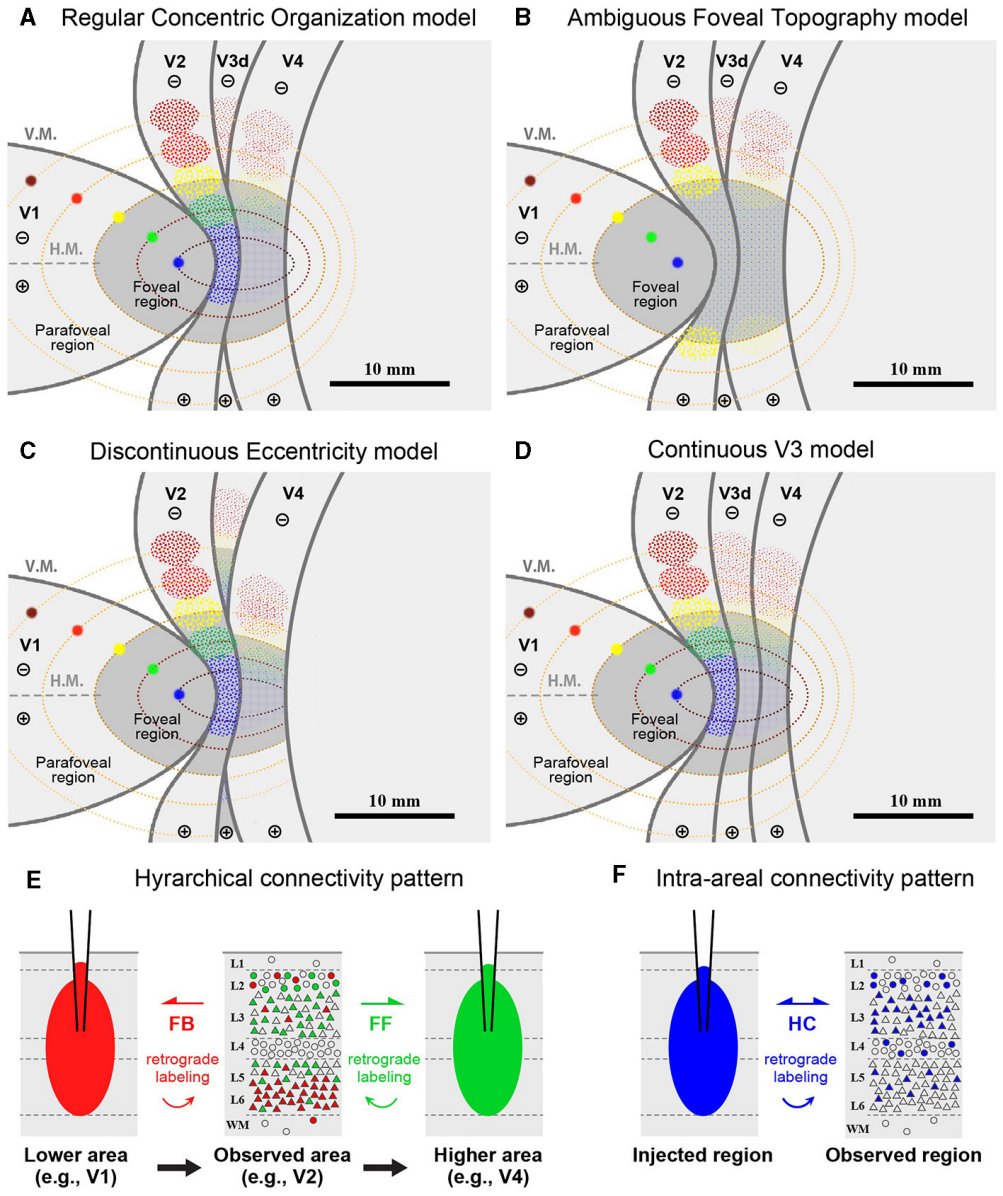
**(A–D)** Hypothetical retrograde labeling patterns following multiple V1 tracer injections based on four different hypotheses. **(A)** If visual areas exhibit a simple extension of isoeccentricity lines across area borders, tracer labeling would align along regular concentric lines. **(B)** In the absence of organized topography in the foveal visual field, tracer labeling would be dispersed around the foveal regions. **(C)** If isoeccentricity lines are discontinuous at area borders, distinct labeling clusters would appear in each visual area with varying locations and sizes. **(D)** If V3 exhibits continuous topography even in the foveal visual field, retrograde labeling clusters would be observed differently in the foveal region compared to V4 clusters, potentially with gaps between them. V.M., vertical meridian; H.M., horizontal meridian; ⊕, upper visual field; ⊖, lower visual field. Scale bars = 10 mm. **(E, F)** Expected differences in layer distribution patterns of retrogradely labeled cells following tracer injections based on inter-areal hierarchical **(E)** and intra-areal horizontal connectivity patterns **(F)**. FB, feedback projection; FF, feedforward projection; HC, horizontal connection.

The topography of the foveal visual field remains a subject of debate, with several competing models proposed. If the topography of visual areas follows a simple continuity along isoeccentricity lines, retrograde labeling from multiple V1 tracer injections would align along regular concentric lines across V1–V4 (Figure 1A). Alternatively, retrograde labeling from foveal V1 may exhibit a scattered distribution around extrastriate areas without specific topography (Figure 1B). This assumption arises from the failure of physiological imaging to reveal retinotopy in the foveal visual areas (Kolster et al., 2009), implying a lack of topographic cortical organization in these regions.

Another model posits that retrograde labeling from V1 may exhibit topographic distribution in V2, V3, and V4, but with discontinuous isoeccentricity lines (Figure 1C). Physiological imaging studies have suggested that V4 may possess smaller retinotopic map than V1 and V2 (Kolster et al., 2009; Arcaro and Kastner, 2015). Additionally, the foveal visual field of V3 may be divided into dorsal and ventral portions (Lyon and Kaas, 2002). Conversely, V3 may be continuous even in the foveal visual field (Figure 1D), a notion supported by imaging studies in the human brain and some previous macaque studies (Wang et al., 2015). If the continuous V3 model holds true, we may find independent clusters of retrogradely labeled cells for the foveal V3 following V1 injections.

Furthermore, we question whether signal distribution patterns follow inter-areal or intra-areal connectivity patterns among V1–V4 in the foveal visual fields (Figures 1E, F). Given the revelation of brain-wide visuotopy, the concept of the “supra-areal eccentricity organization model” has gained traction (Arcaro and Kastner, 2015). This model suggests large-scale coherence in the visuotopic organization across the cortex, emphasizing local continuity across individual visual field maps (Buckner and Yeo, 2014). If this concept holds true, connectivity patterns in the foveal visual field may adhere to intra-areal connectivity patterns, where there is no distinction between feedback and feedforward, and projections are horizontally interconnected in a wide variety of layers, including layer 4, while regular feedback projections predominantly arise from infragranular layers (Lund, 1988; Felleman and Van Essen, 1991; Barone et al., 2000; Markov et al., 2014; Rockland, 2022; Shamir and Assaf, 2023).

In this study, we injected a small volume of four types of retrograde tracers focally, encompassing the foveal and parafoveal regions of V1 in two macaques, and studied connectivity with extrastriate visual areas to address the questions outlined above. Additionally, we injected them into foveal and parafoveal regions of V4 and studied connectivity with earlier visual areas in two other macaques. Three of the four types of tracers were cholera toxin subunit B (CTB) with different fluorescent tags, expecting them to behave similarly in live animals, enabling more convincing topographic studies than previous efforts. Our results demonstrate that the topography of foveal V3 and V4 is not simply an extension of the eccentricity representations of V1 and V2 but is more concentrated than the topography of V1 and V2.

## Materials and methods

### Animals

The materials and methods essentially followed those described previously (Liu et al., 2021) with minor modifications. Information about each monkey is summarized in Table 1. Four adult rhesus macaque monkeys (*Macaca mulatta*) were utilized in this study. Retrograde tracers, including cholera toxin subunit B conjugated with Alexa Fluor 488 (CTB-488; Thermo Fisher Scientific, MA), CTB conjugated with Alexa Fluor 555 (CTB-555; Thermo Fisher Scientific), CTB conjugated with Alexa Fluor 647 (CTB-647; Thermo Fisher Scientific), and biotinylated dextran amine 3,000 kDa (BDA; Thermo Fisher Scientific), were employed. Case 1 and Case 4 were previously subjected to separate electrophysiology studies in the motor cortex, and all four cases were involved in a distinct neuronal tracing study with CTBs and BDA injected into the prefrontal cortex of the other hemispheres. However, we consider that these treatments did not influence the results of the current study.

**Table 1:** Summary of monkeys used in this study.

**Case information**			**Injection information**					**Fixation**
**Animal**	**Gender**	**Body weight (kg)**	**Site**	**Tracer**	**Volume (nl)**	**Injection regions**	**Survival period after injections (days)**	**PFA concentration for fixation (%)**
Case 1	Male	9.8	No. 01 No. 02 No. 03 No. 04	CTB-647 CTB-488 CTB-555 BDA	70 × 2 70 × 2 70 × 2 50 × 2	Right hemisphere, V1	14	2%
Case 2	Female	3.9	No. 05 No. 06 No. 07 No. 08	CTB-555 CTB-488 CTB-647 BDA	50 × 2 50 × 2 50 × 2 40 × 2	Right hemisphere, V1	23	1%
Case 3	Male	10.0	No. 09 No. 10 No. 11 No. 12	CTB-555 CTB-647 CTB-488 BDA	70 × 2 70 × 2 100 × 2 70 × 2	Left hemisphere, V4	13	2%
Case 4	Male	9.5	No. 13 No. 14 No. 15 No. 16	CTB-647 CTB-488 CTB-555 BDA	70 × 2 100 × 2 70 × 2 70 × 2	Right hemisphere, V4	14	2%

### Surgery and tracer injections

Anesthesia was induced intramuscularly with ketamine (10–30 mg/kg body weight, i.m.) and maintained by 0.5%−2.0% isoflurane inhalation during surgery. The animals were positioned in a stereotactic frame surrounded by a heating pad to maintain their body temperature at around 37°C. Atropine (0.10 mg/kg body weight, i.m.) and dexamethasone (0.25 mg/kg body weight, i.m.) were administered to reduce mucous secretion and brain edema, respectively. Anesthetic depth was continuously monitored, including heart rate, exhaled CO_2_, SpO_2_, and respiratory rates. Intravenous saline and glucose saline were administered to maintain hydration and provide energy throughout the surgery. Lidocaine was subcutaneously administered to alleviate pain during skin incision.

After the craniotomy, the dura was opened to expose landmarks such as the lunate sulcus (lus), inferior occipital sulcus (ios), and superior temporal sulcus (sts). We aimed to inject the foveal representations of V1 in Case 1 and Case 2, and V4 in Case 3 and Case 4. The injection sites were determined based on previous imaging studies and anatomical studies (Maguire and Baizer, 1984; Gattass et al., 1988; Nakamura et al., 1993; Brewer et al., 2002; Kolster et al., 2014), which were confirmed by subsequent histological examinations. During the injection, the brain surface was covered with an artificial dura to suppress cortical pulsation, except at the injection sites. A Hamilton syringe with a glass pipette (tip size: 75–150 μm in diameter) was used to pressure-inject 40–100 nl of the tracer (1 mg/ml for CTB, 100 mg/ml for BDA) at a rate of 30–45 nl/min. We positioned the injection pipettes as perpendicular to the cortical surface as possible, then inserted them 2.0 mm deep into the brain. After moving up 0.2 mm, we made the first injection, followed by moving up 1.0 mm and making the second injection. This procedure ensured the creation of a pocket for the diffusion of tracer chemicals. The pipette was left in place for 5 min before retraction.

Following the injection, the artificial dura and the original skull were employed to cover the brain surface, filling the gap with dental cement. The skin was sutured, and lidocaine hydrochloride gel was applied to the scalp. Buprenorphine (0.005–0.010 mg/kg body weight, i.m.) and cephalosporin (20 μg/kg body weight, i.m.) were administered to alleviate pain and prevent inflammation. The entire surgery lasted 7–15 h. After the animals regained spontaneous breathing, they were returned to their home cages. Buprenorphine and cephalosporin were continuously administered for at least 3 days, and more fruit and toys were provided than usual after surgery to aid in the animals' quick recovery.

### Histological procedures

After a survival period of 2–3 weeks, all animals were again anesthetized using ketamine (10–30 mg/kg body weight, intramuscularly). Once sedated, they were subjected to a pentobarbital overdose (50–100 mg/kg body weight, i.v. or intraperitoneally). Deep anesthesia was confirmed with the loss of reflexes, after which they were transcardially perfused with phosphate buffer (PB), followed by a mixture of 1%−2% paraformaldehyde (PFA) and 10% sucrose in PB. Subsequently, we removed the entire brain from the skull, promptly isolated the visual cortices from the rest of the brain, and flattened them, following previous studies (Stepniewska and Kaas, 1996; Sincich et al., 2003). The flattened visual cortices were sandwiched between glass slides and immersed overnight in a 30% sucrose/PB solution at 4°C. The cortices were then tangentially sectioned at 40 μm using a freezing microtome (YAMATO KOKI, Tokyo, Japan). All sections were preserved at −20°C in a cryoprotectant solution [30% ethylene glycol, 30% glycerol, and 40% phosphate-buffered saline (PBS)] until used. We divided the sections into six series, each containing every six sections, and allocated three series of them for BDA staining. We scanned them for dark field fluorescence, with two series of them reserved for further counterstaining with cresyl violet. Two series of the remaining three were reserved for cytochrome oxidase (CO) staining, and one series was kept as a backup.

For BDA staining, sections were first rinsed twice with 0.3% Triton X-100 in PBS (PBST, pH 7.4). BDA was then visualized using the standard avidin-biotin-peroxidase method (Vectastain ABC kit, Vector Laboratories, Burlingame, CA) according to the manufacturer's instructions. The floating sections were incubated for 5–10 min in a reaction buffer containing 100 μg/ml 3,3′-diaminobenzidine (DAB; Sigma-Aldrich, St. Louis, MO), 100 μg/ml nickel chloride, and 0.01% H_2_O_2_ in PBST. Sections were rinsed three times with PBST between each reaction step, mounted on glass slides, coverslipped with aqueous glue, and scanned using both bright-field and dark-field fluorescent microscopy (VS-120, Olympus, Tokyo, Japan).

For sections undergoing cresyl violet counterstaining following BDA staining, coverslips were removed, and sections air-dried for several days. Sections were rinsed sequentially in distilled water, 90% ethanol, and 75% ethanol. They were then stained with a 0.1% cresyl violet solution for 5–10 min. Excess cresyl violet was washed off with a 0.8% acetic anhydride solution in 90% ethanol for 5–10 min. After dehydration in increasing concentrations of ethanol, sections were immersed in xylene, and coverslipped with xylene-based glue for bright field scanning.

For CO staining, floating sections were rinsed twice with 5% sucrose in PBS. They were incubated in a CO reaction buffer [consisting of 200 μg/ml cytochrome C (Sigma-Aldrich, St. Louis, MO), 150 μg/ml catalase (Sigma-Aldrich), and 100 μg/ml DAB (Sigma-Aldrich) in 5% sucrose/PBS] for 5–18 h. The reaction was stopped with 5% sucrose/PBS. After mounting on glass slides, the sections were dehydrated through a graded series of ethanol, immersed in xylene, and coverslipped with xylene-based glue.

### Data analysis

Images were processed using Olympus VS-120 software, Adobe Photoshop, and Adobe Illustrator (CC 2018, Adobe, San Jose, CA). We integrated three fluorescence channels from the dark field and manually distinguished neurons based on their color and the structure of their cell bodies against the background. Neurons positive for green, red, and blue corresponded to CTB-488, CTB-555, and CTB-647, respectively. For BDA-labeled neurons, we identified them by their brown color and cell body structure in both the bright and dark field. Vasculature patterns were utilized to precisely align sections in both the bright and dark field, including CO and cresyl violet (Nissl) sections, facilitating region and laminar classifications. To study laminar differences, we divided the labeling into three layers: supragranular, middle, and infragranular based on counter cresyl violet staining and adjacent CO staining. Supragranular layers included layers 1–3, and infragranular layers included layers 5 and 6. Middle layers mainly included layer 4 but also encompassed some of deeper layer 3 and upper layer 5 due to challenges in precisely demarcating layers in tangential sections. It's worth noting that layer numbering in V1 may exhibit inconsistency across researchers (Balaram et al., 2014). In this manuscript, “middle layers” encompass Brodmann's layers 4A, 4B, and 4C, which are Hässler's layers 3Bβ, 3C, and layer 4. After plotting all labeled neurons in each section and aligning the sections, we used customized Python programs (Python 3.0, Wilmington, Delaware) to analyze the number and location of these neurons. R packages (v 4.3.1, R Foundation for Statistical Computing, Vienna, Austria) were employed to represent the density of different labels, including overlapping areas not shown.

To assess layer difference in signal distribution, we calculated the percentage of labeled neurons for each tracer, using the number of neurons in the target region as the numerator and the total number of neurons in V1, V2, V3, and V4 (excluding injection sites) as the denominator. For example, in the foveal V1 injection cases (Case 1 and Case 2), the number of labeled neurons in the given layer (supragranular, middle, or infragranular layer) of the target region (foveal V2 or foveal V4) was divided by the number of labeled neurons in all layers of V2, V3, and V4, not including intra-areal projection neurons.

Arithmetic mean coordinates were used to locate the center of labeled neurons in different regions. Only the group of labeled neurons in that area, which was more than 5%, was selected for analysis. However, for Case 4, due to minimal and overly sparse CTB-488, CTB-555, and BDA labeling in V1, we did not locate the centers of these tracers in V1. The equation used was:


$$\begin{array}{l} p_{center}=\left(x_{center},y_{center}\right) \end{array}$$

$$\begin{array}{l} x_{center}=\frac{1}{n}\overset{n}{\underset{i=1}{\sum }}x_{i} \end{array}$$

$$\begin{array}{l} y_{center}=\frac{1}{n}\overset{n}{\underset{i=1}{\sum }}y_{i} \end{array}$$

$$\begin{array}{l} d_{center}=\;\sqrt{{\left(x_{center1}-x_{center2}\right)}^{2}+{\left(y_{center1}-y_{center2}\right)}^{2}} \end{array}$$

Each case had its own baseline distance for injection sites. The positions of injection sites were identified in the middle sections of each case followed by calculating the distance of adjacent injection sites' centers. We then calculated the scaling value of the neighboring centers relative to the injection sites of these two tracers by logarithm. If the scaling value was >0.0, the distance between neighboring centers was considered greater than that of the injection sites. The equation used was:


$$\begin{array}{l} scaling\;rate=\log_{10}\left(\frac{d_{center}}{d_{injection}}\right) \end{array}$$

## Results

### Distribution of labeled neurons in V1 injection cases

#### Case 1

**Figure 2:**
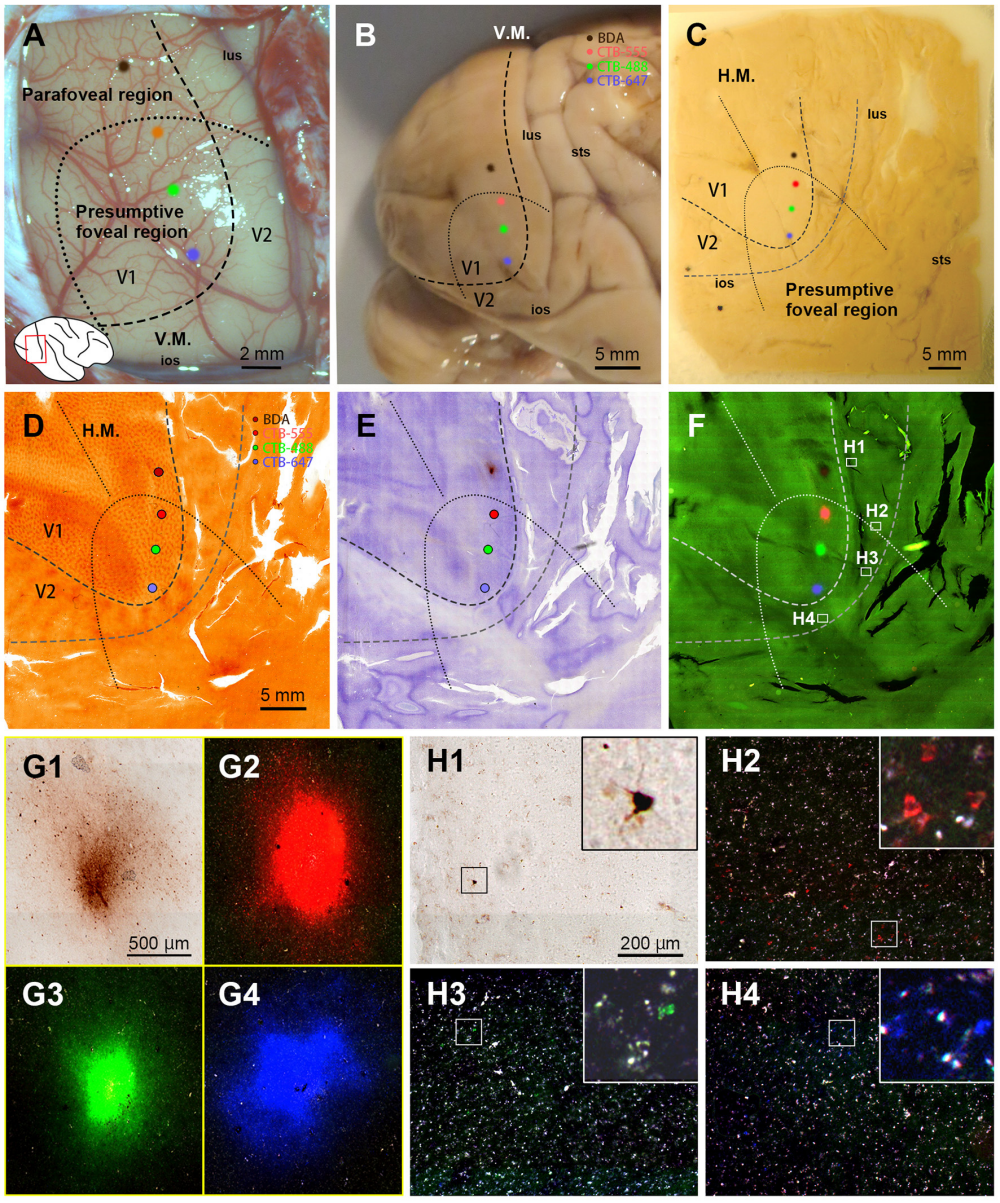
Retrograde tracer injections in Case 1. **(A)** Foveal V1 injection sites imaged at the time of tracer injection (blue: CTB-647; green: CTB-488; red: CTB-555; brown: BDA) with presumed V1/V2 border and foveal region border. Right is anterior and upper is dorsal; **(B)** Macaque brain photograph depicting presumed V1/V2 border, foveal region border, and injection sites immediately after extraction; **(C)** Flattened brain tissue photograph with presumed V1, V2 borders, horizontal meridian, foveal region border, and injection sites; **(D–F)** Tangential sections of flattened V1 stained for CO activity **(D)**, BDA labeling with Nissl substance counterstaining **(E)**, and dark field image of fluorescent labeling **(F)**; **(G1–G4)** Higher magnification of four injection sites from the section in **(F)**; **(H1–H4)** Higher magnification of real fluorescent labeling in framed regions in **(F)**. Retrogradely labeled neurons are even more magnified in insets; lus, lunate sulcus; ios, inferior occipital sulcus; sts, superior temporal sulcus; H.M., horizontal meridian; V.M., vertical meridian. Scale bars in **(D)**, **(G1)**, and **(H1)** are applied to **(D–F)**, **(G1–G4)**, and **(H1–H4)**, respectively.

In this case, we targeted the foveal to parafoveal V1 region sequentially with CTB-647 (blue), CTB-488 (green), CTB-555 (red), and BDA (brown). During injection, V1 borders were estimated by transitioning from a vessel-dense region (presumably V1) to a vessel-pale region (presumably extrastriate visual areas). This border was considered the vertical meridian (V.M.) representation (Figures 2A, B). Referring to retinotopic mapping studies (Van Essen et al., 1984; Sincich et al., 2003; Kolster et al., 2009; Takahata et al., 2018), the horizontal meridian (H.M.) representation was estimated as the long axis of the V1 ellipse, and the foveal visual field was estimated within 10 mm from the anterior edge of the V1 ellipse. Dorsal and ventral regions from the H.M. were considered the lower and upper visual quadrants, respectively. Based on these estimations, CTB-647 was injected near the vision center (the anterior edge of the V1 ellipse), and CTB-488, CTB-555, and BDA were injected about 3 mm apart, each targeting to align parallel to the V.M. The four injection sites were carefully plotted by dots with corresponding colors based on recorded movies during injections and anatomical landmarks (Figures 2A, B).

**Figure 3:**
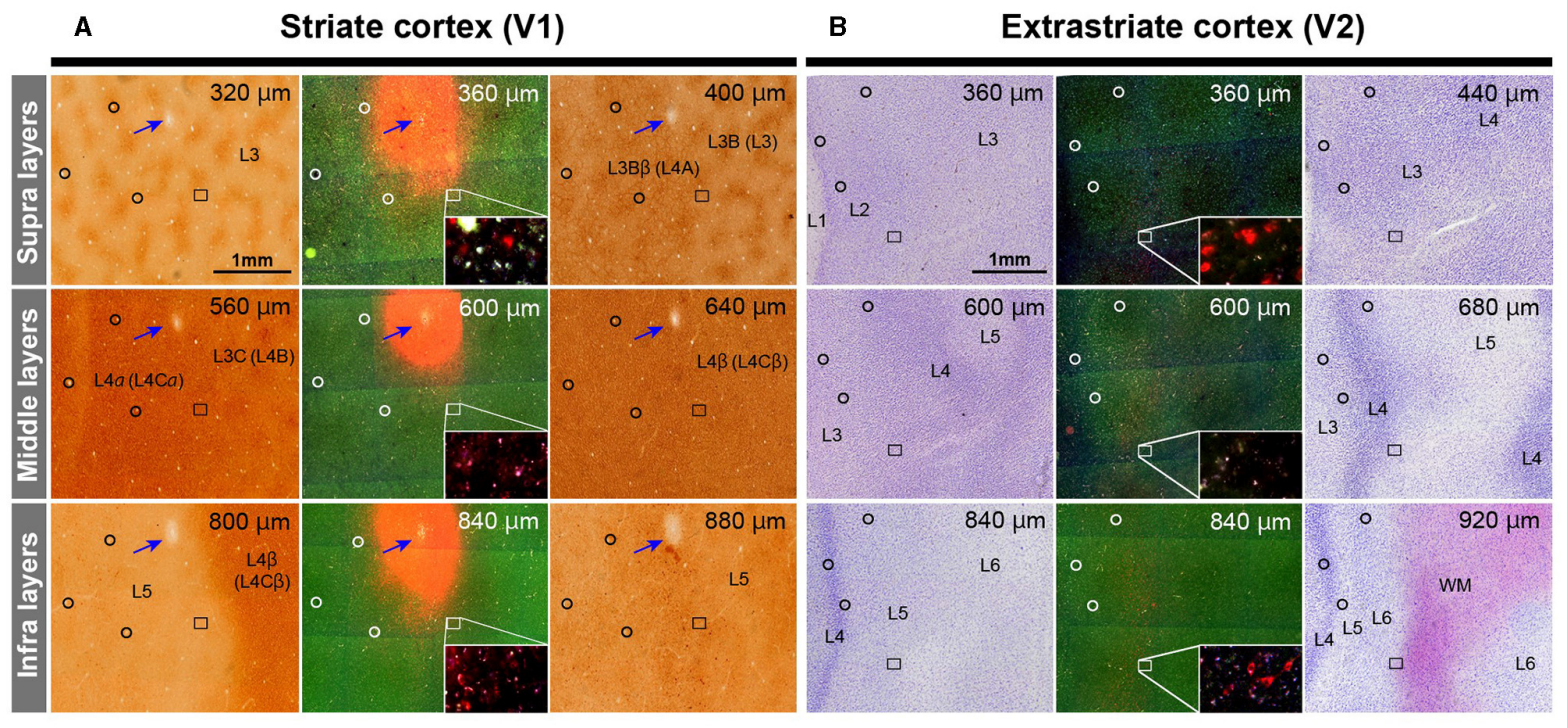
Illustrations for layer divisions. **(A)** Tangential sections stained for CO (left and right columns), and dark field fluorescence (middle column) of Case 1 are shown. Upper, middle, and bottom panels are for supragranular, middle, and infragranular layers, respectively. Images are aligned in the guidance of vasculature patterns (black circles). Even though each section contained several layers due to incomplete flattening, layers were identified by CO staining patterns in V1. Blue arrows indicate the injection site of CTB-555 identified by the loss of CO staining, robust fluorescence, and a consecutive hole in the tissue. The rectangle area is magnified in the inset to exhibit retrograde labeling of cells. Layer number are indicated by L3, L3B (L3), L3Bb (L4A), L5 and L6. Here, we recruit Hässler's scheme for V1 layer numbering, and the numbering by Brodmann is written in the parenthesis (Balaram et al., 2014). **(B)** Tangential sections stained for Nissl substance (left and right columns), and dark field fluorescence (middle column) of Case 1 are shown. Layers were identified by Nissl staining patterns in extrastriate areas. Upper right numbers represent depth from the pial surface. Scale bar = 1 mm.

After brain removal, we unfolded sulci, dissected white matter, and flattened visual areas, documenting morphological relationships among injection sites, sulci locations, and presumed retinotopic maps of visual areas by taking photos at each step (Figure 2C). Tangential sections were cut at 40 μm for CO staining, BDA staining, Nissl staining, and dark field scanning (Figures 2D–F). The sizes of intense labeling for the injection sites were ~0.5–1.0 mm in diameter (Figures 2G1–2G4). Scattered retrograde labeling of tracers was abundant in V2 (Figures 2H1–2H4). In the flattened images, V1 extension and layers were characterized by blobs in layers 2/3, and intense signal of CO in layer 4 (Figure 3A), and V2 extension was identified by characteristic stripe patterns in CO staining (Livingstone and Hubel, 1982; Tootell et al., 1983; Horton and Hocking, 1998; Sincich et al., 2003). Nissl staining revealed layer 4 by intense labeling, and layer 1 and white matter by pale labeling (Figure 3B). Injection sites were identified by dense fluorescent labeling and BDA signals, as well as the loss of CO signal with penetration holes (blue arrows in Figure 3A).

**Figure 4:**
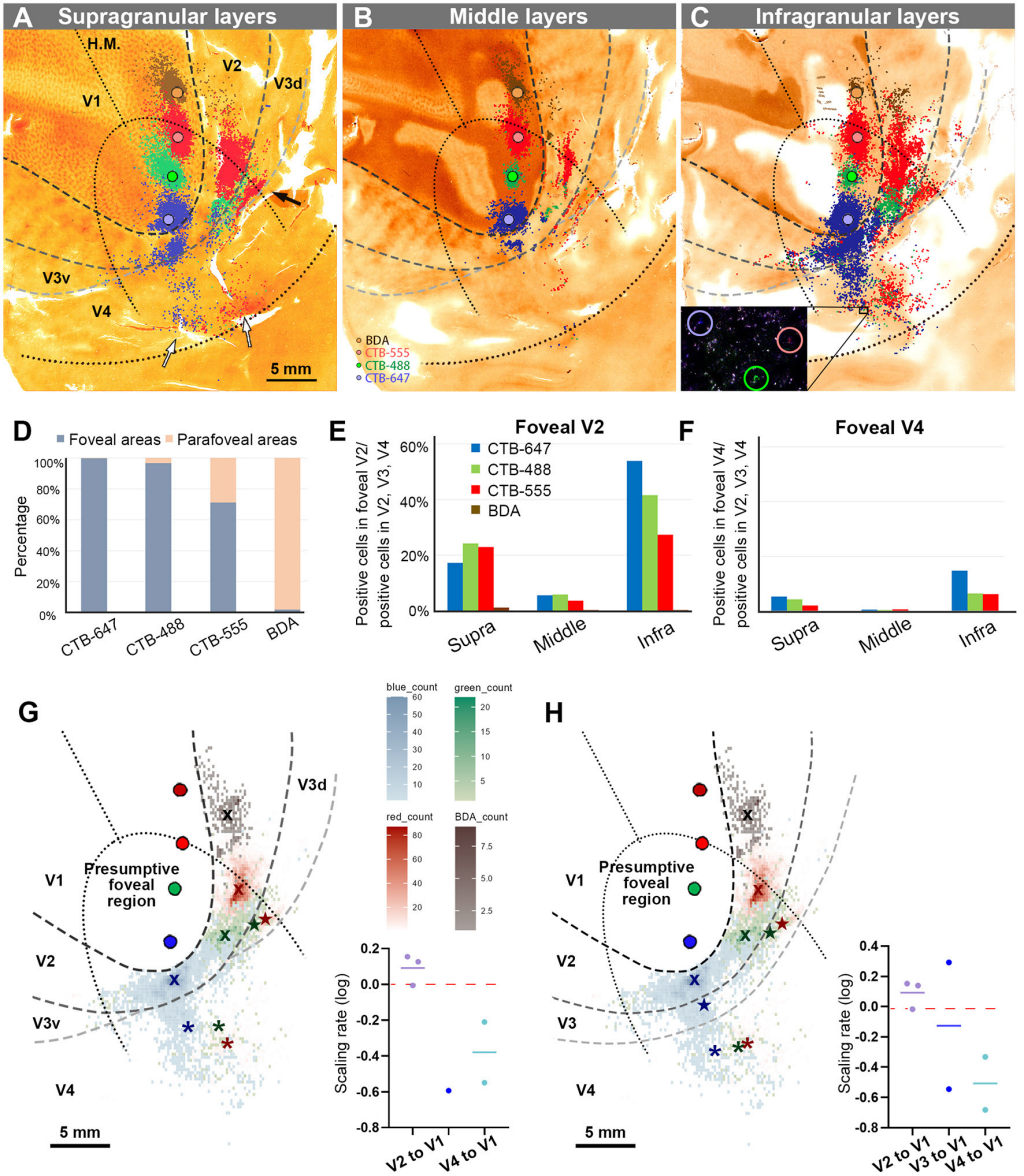
Retrograde labeling in Case 1. **(A–C)** All labeled cells are plotted and separated into supragranular **(A)**, middle **(B)**, and infragranular layers **(C)**. Plots are overlaid on flattened visual cortex stained for CO activity. Colored dots represent labeled cells (blue: CTB-647, green: CTB-488, red: CTB-555, and brown: BDA). Examples of labeled cells are circled for each color in high magnification in the inset in **(C)**. A black arrow indicates clusters in V3d, and white arrow indicate clusters in V4; **(D)** Percentages of distribution of labeled neurons based on their foveal and parafoveal localizations; **(E, F)** Percentages of distribution of labeled neurons located in different layers in foveal V2 **(E)**, and foveal V4 regions **(F)**, shown out of all labeled neurons in V2, V3 and V4; **(G)** An illustration of labeling density, and scaling rates of cluster centers in V2, V3, and V4 relative to the distance of adjacent injection sites in V1, based on the hypothetical discontinuous V3 model. “x”, star, and asterisks indicate cluster centers in V2, V3, and V4, respectively; **(H)** An illustration of labeling density, and scaling rates of cluster centers in V2, V3 and V4 relative to the distance of adjacent injection sites in V1 based on the hypothetical continuous V3 model.

The receptive field of the CTB-647 injection site was slightly upper visual field near the vision center, while those of CTB-488 and CTB-555 were in the lower visual field within the foveal visual field. The BDA injection site was right outside the foveal visual field. Images were carefully demarcated into three layers of supragranular (layers 1–2, and upper layer 3), middle (deeper layer 3, layer 4, and upper layer 5), and infragranular layers (layers 5–6), and every labeled neuron was manually plotted in high magnification images (Figures 4A–C). V3 borders were speculated recruiting the discontinuous model (Lyon and Connolly, 2012), referring slightly darker CO staining patterns (Lyon and Kaas, 2001; Sincich et al., 2003). The signals spread into V2, V3, and V4, as well as V1 near the injection sites. V2 contained the majority of labeled neurons, appearing clustered near the injection sites for each color. Retrograde signals were abundant in supragranular and infragranular layers, but less in middle layers. Immediately outside V2, another group of clusters for CTB-488 and CTB-555 was observed (indicated by a black arrow in Figure 4A), likely in V3d. Furthermore, scattered signals in a more ventral anterior part (indicated by white arrows in Figure 4A) were most likely in V4. To statistically evaluate possible topographic distribution of labeled neurons, we counted the number of labeled neurons in extrastriate visual areas between the foveal and parafoveal visual fields. A half-ellipse line of presumptive foveal visual field was drawn at ~10 mm from the edge of the V1 ellipse as shown in Figures 4A–C. Lines of area borders among V1, V2, V3d, V3v, and V4 were also drawn.

According to our analysis, 99.6% of CTB-647 labeled cells, 96.6% of CTB-488 labeled cells, and 71.2% of CTB-555 labeled cells were found in the foveal visual field. For BDA labeled cells, the parafoveal visual field covered 98.0% of them (Figure 4D). The results suggest that the closer the injection site is to the vision center (the edge of the V1 ellipse), the more labeled neurons are distributed in the foveal visual field, implying that the topographic map is maintained from V1 to extrastriate visual areas.

To assess differences across layers, we counted labeled cells in the foveal V2 and V4 for each tracer, calculating the percentages of these cells among all labeled cells in V2, V3, and V4 (see methods; Figures 4E, F). Due to rare BDA signals in the foveal visual field, percentages of labeled cells in the foveal V2 and V4 were nearly zero. However, we obtained sufficient ratios for the other three tracers. Infragranular layers dominated percentages over supragranular and middle layers in both foveal V2 and V4 regions. In supragranular layers of V2, percentages of CTB-647, CTB-488, and CTB-555 labeled cells were 17.3%, 24.3%, and 22.9%, respectively. In infragranular layers of V2, they were 53.9%, 41.6%, and 27.4%. In middle layers of V2, they were 5.7%, 5.9%, 3.7%. In infragranular layers of V4, they were 14.7%, 6.3%, and 6.1 %, while in supragranular layers of V4, they were 5.2%, 4.2%, and 2.0%, and in middle layers of V4, they were 0.4%, 0.4%, and 0.6%. These results suggest that feedback projections from foveal V2 and V4 predominantly arise from neurons in infragranular layers, rather than supragranular or granular layer 4 to foveal V1 neurons.

Cluster-like signal distribution patterns observed in extrastriate visual areas prompted us to determine cluster center locations. We calculated central position coordinates using the arithmetic mean of Cartesian coordinates of the same type of labeling in a specific visual area. Cluster center locations in relation to injection sites and cortical topography are shown in Figure 4G. Cluster centers in V2 aligned with the injection sites in V1 and appeared on the same isoeccentricity lines as V1. However, in V4, cluster centers were scattered near the V1 edge. We also calculated scaling rates between injection sites and cluster centers. The scaling rates between CTB-647 and CTB-488, CTB-488 and CTB-555, CTB-555 and BDA were nearly zero in V2 (0.13, −0.01, and 0.15, respectively), indicating almost comparable topography in V2 than in V1. In V3 and V4, however, the scaling rates were lower than zero (−0.59, −0.21, and −0.55 for the scaling rates between CTB-488 and CTB-555 in V3, between CTB-647 and CTB-488 in V4, and CTB-488 and CTB-555 in V4, respectively), indicating that the topography of the foveal V3 and V4 is much smaller than that in the foveal V1. Since the labeling of BDA was too few in V3 and V4, we did not determine cluster centers for BDA in V3 or V4. These results suggest that the topography is mostly continuous from the foveal V1 to the foveal V2, but not to the foveal V3 and V4, and the map is more concentrated in the foveal V3 and V4.

To examine statistics based on the continuous V3 model, we drew the border of the hypothetical continuous V3, marked cluster centers, and calculated the scaling rates (Figure 4H). The results and conclusions were mostly similar, except that the scaling rate of CTB-647 and CTB-488 was large in V3 (0.29).

**Figure 5:**
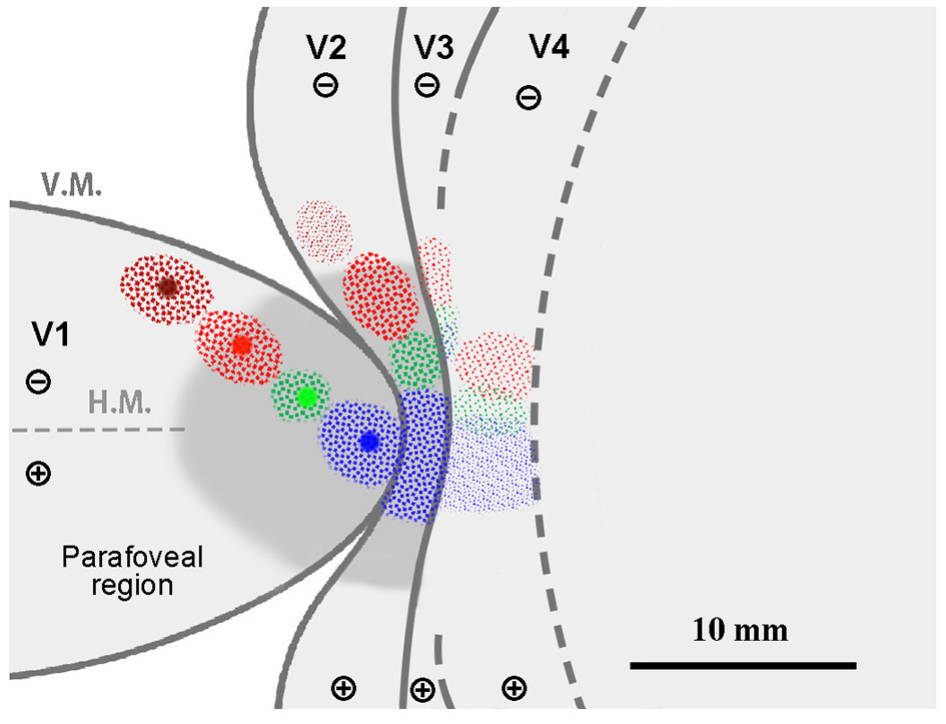
A simplified illustration of injections and retrograde labeling in the visual cortex relative to presumed cortical map in Case 1.

To summarize the results of Case 1 and for ease of comparison with subsequent cases, we illustrated signals on a simplified map of retinotopy and visual areas in Figure 5. Retrograde labeling clusters were observed along the same scaling rates as the injection sites in V2, but they were more concentrated in V4, although they maintained topographical alignment. Almost no signals were observed for BDA in V4, which was injected into the parafoveal V1. For CTB-488 and CTB-555, another group of clusters was observed in presumptive V3d. The cluster of CTB-647 is large anterior to V2, and the separation between V3 and V4 was unclear.

#### Case 2

**Figure 6:**
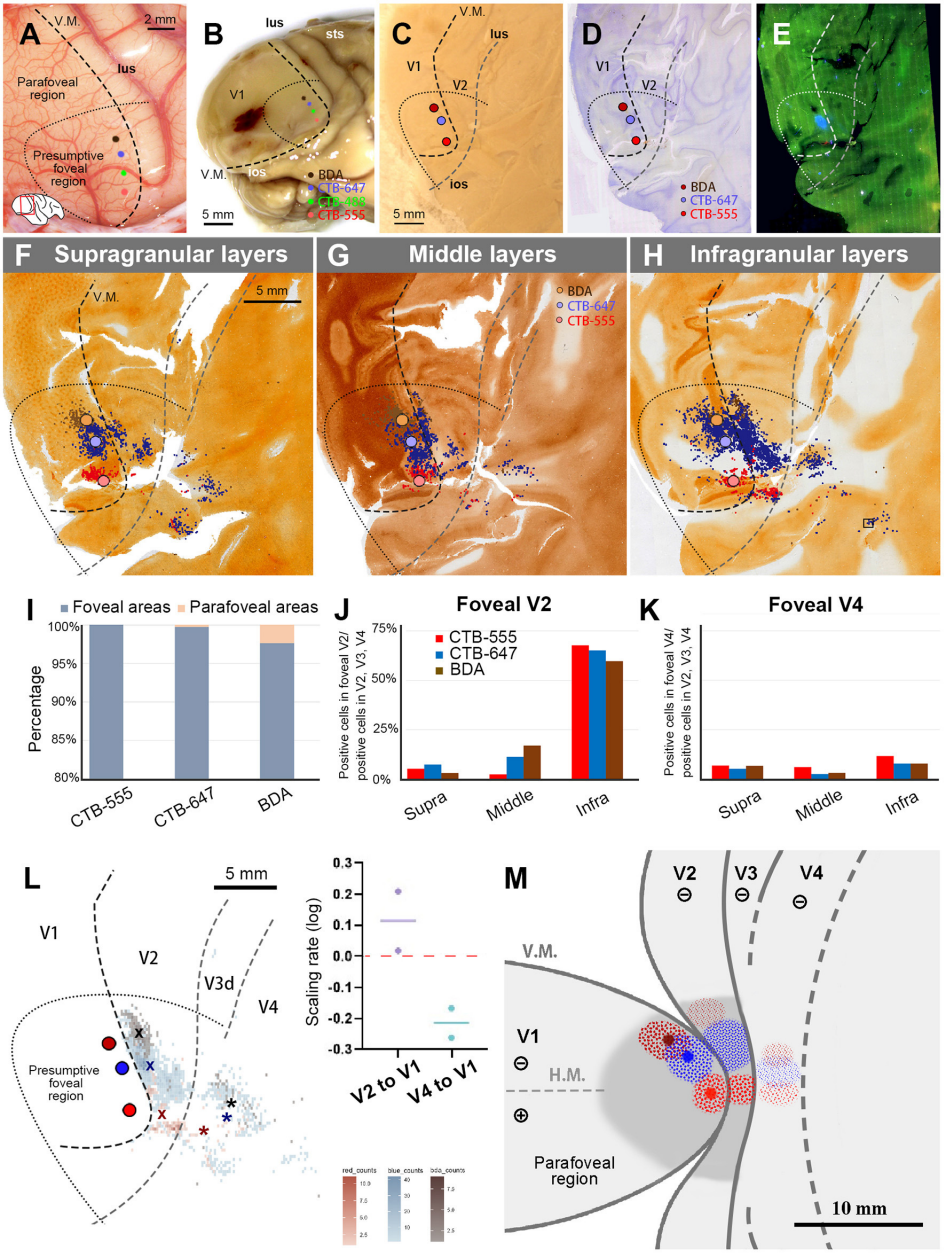
Tracer injections and retrograde labeling in Case 2. **(A)** An image of the foveal V1 at the time of injection with injection sites (red for CTB-555; blue for CTB-647; brown for BDA), and presumed V1/V2 border, and foveal region border. Right is anterior and upper is dorsal; **(B)** Macaque brain photograph depicting injection sites, presumed V1/V2 border, and foveal region border immediately after extraction; **(C)** Flattened brain tissue photograph with injection sites, V1 border, V2 border, and foveal region border; **(D, E)** Tangential sections of flattened V1 stained for BDA labeling with Nissl substance counterstaining **(D)**, and dark field image of fluorescent labeling **(E)**; **(F–H)** All labeled cells are plotted and separated into supragranular **(F)**, middle **(G)**, and infragranular layers **(H)**. Plots are overlaid on flattened visual cortex stained for CO activity. Colored dots represent labeled cells. Examples of labeled cells are shown in high magnification in the inset in **(H)**; **(I)** Percentages of distribution of labeled neurons based on their foveal and parafoveal localizations; **(J, K)** Percentages of distribution of labeled neurons located in different layers in foveal V2 **(J)**, and foveal V4 regions **(K)**, shown out of all labeled neurons in V2, V3 and V4; **(L)** An illustration of labeling density, and scaling rates of cluster centers in V2 and V4 relative to the distance of adjacent injection sites in V1. “x” and asterisks indicate cluster centers in V2 and V4, respectively; **(M)** A simplified illustration of injections and retrograde labeling in the visual cortex relative to presumed cortical map.

In this case, we altered the sequence of four tracers from the foveal V1 region to the parafoveal V1 region, namely CTB-555, CTB-488, CTB-647, and BDA. We followed the same criteria to estimate the topographic organizations during the injection as Case 1 (Figures 6A, B). CTB-555 was injected in the vicinity of the vision center, and CTB-488, CTB-647, and BDA were injected about 2.0 mm apart, each targeting alignment parallel to V.M. After a 23-day survival period, however, tissue compression due to brain inflammation posed challenges in unfolding sulci during flattening. Despite these difficulties, we applied the same flattening and sectioning procedures (Figure 6C), along with histology works using CO staining, BDA staining, and Nissl staining (Figures 6D, E). We observed signals of CTB-555, CTB-647, and BDA, but not CTB-488, likely due to the failure of CTB-488 injection. Scattered retrograde labeling of tracers was clearly observed, and we manually plotted every labeled neuron, categorizing the labeled neurons into the same three depth layers and foveal/parafoveal visual fields as in Case 1 (Figures 6F–H). Signals spread into V2 and V4, as well as V1 near the injection site. Outside V1, the majority of labeled cells were found in V2, appearing as clusters near the injection sites for each tracer type. According to our statistical analysis, 100% of CTB-555 labeled cells, 99.7% of CTB-647 labeled cells, and 97.6% of BDA labeled cells were found in the foveal visual field (Figure 6I). The labeling in the extrastriate visual areas exhibited a similar distribution, maintaining the topographic map in V1, despite changing the sequence of injections, consistent with Case 1.

We also separately counted the number of labeled cells in the foveal V2 and V4, calculating the percentages of them among all labeled cells in V2, V3, and V4 (Figures 6J, K). Although brain inflammation may have caused some loss of labeled neurons, we still obtained sufficient numbers of labeled cells for the three tracer types. According to our analysis, infragranular layers were dominant in percentages over the other two types of layers in both foveal V2 and V4. In infragranular layers of V2, the percentages of CTB-555, CTB-647, and BDA labeled cells were 67.7%, 65.2%, and 59.7%. In infragranular layers of V4, they were 11.7%, 7.9%, and 7.9%, while they were 6.9%, 5.3%, 6.7% in supragranular layers and 6.0%, 2.6%, 3.2% in middle layers. These results suggest that feedback projections from the foveal V2 and V4 to V1 predominantly originate from neurons in infragranular layers, as observed in Case 1.

The cluster center locations of CTB-555, CTB-647, and BDA were all in the lower visual quadrant (Figure 6L). In V2, they aligned just like the injection sites in V1, with the locations of the cluster centers appearing on the same isoeccentricity lines as the injection sites. However, the cluster centers were scattered near the V1 edge in V4. We also calculated the scaling rate between the injection sites and the cluster centers. In our analysis, the scaling rates between CTB-555 and CTB-647, and CTB-647 and BDA were 0.02 and 0.21, respectively, in V2, whereas in V4, the scaling rates between CTB-555 and CTB-647, and CTB-647 and BDA were lower than zero (−0.26 and −0.17, respectively). These results suggest that the topographic map is mostly continuous from the foveal V1 to the foveal V2 but not to the foveal V4, and the map is more focused in the foveal V4, consistent with Case 1.

Figure 6M shows a simplified map of retinotopy with our tracing results for Case 2. For ease of comparison, we use the same map as Figure 5 here. Retrograde labeling clusters were observed along the same isoeccentricity lines as the injection sites in V2, but they were more concentrated in V4, although they maintained topographical alignment.

### Distribution of labeled neurons in V4 injection cases

#### Case 3

**Figure 7:**
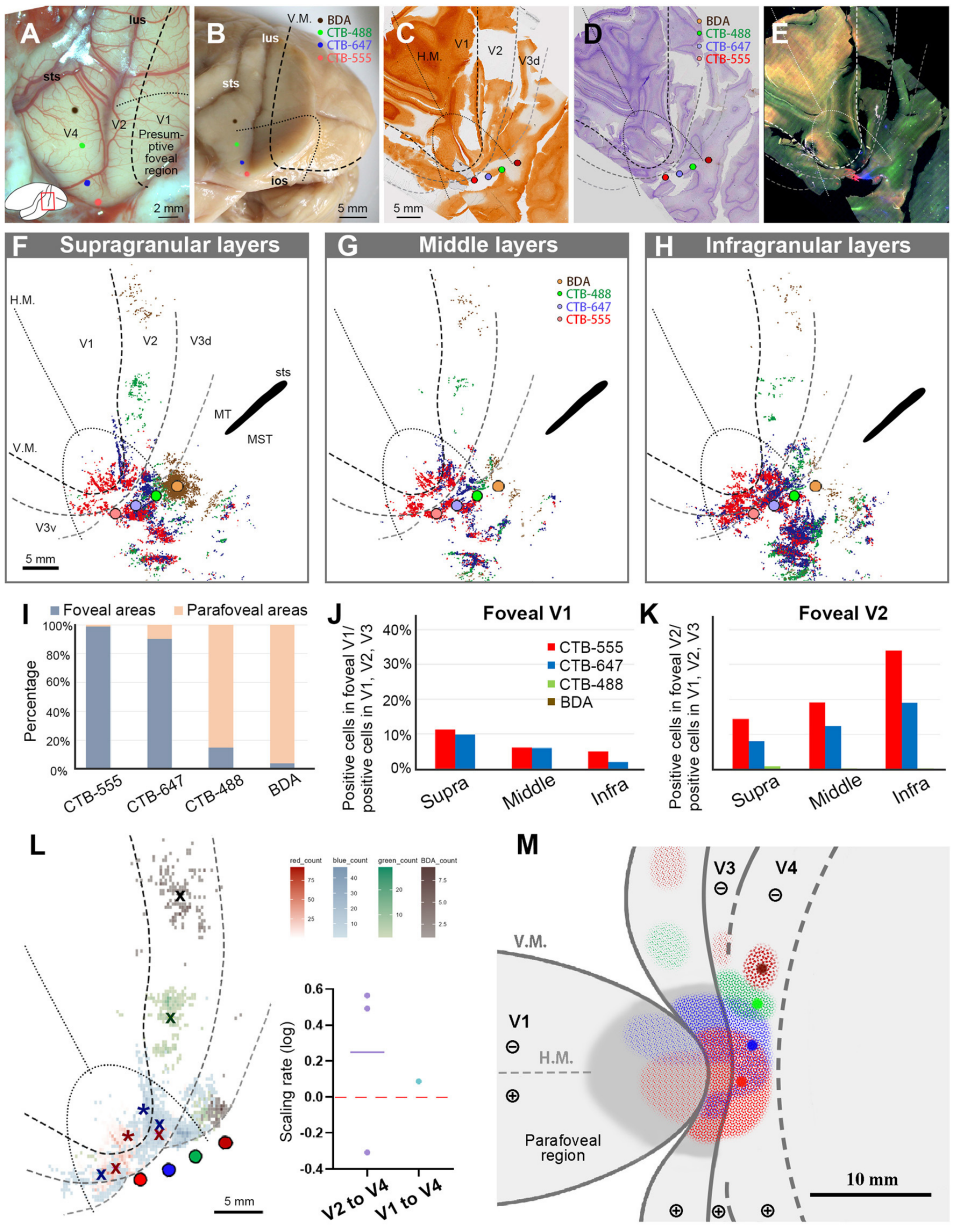
Tracer injections and retrograde labeling in Case 3. **(A)** An image of the foveal V1 at the time of injection with injection sites (red for CTB-555; blue for CTB-647; green for CTB-488; brown for BDA), and presumed V1V2 border and foveal region border. Left is anterior and upper is dorsal; **(B)** Macaque brain photograph depicting injection sites, V1/V2 border, and foveal region border, immediately after extraction; **(C–E)** Tangential sections of flattened V1 stained for CO activity **(C)**, BDA labeling with Nissl substance counterstaining **(D)**, and dark field image of fluorescent labeling **(E)**. This cortex is the left hemisphere, but right and left is inverted for ease of comparison with other cases; **(F–H)** All labeled cells are plotted and separated into supragranular **(F)**, middle **(G)**, and infragranular layers **(H)** in the map of flattened visual cortex. Colored dots represent labeled cells. MT/middle temporal area; MST/middle superior temporal area; **(I)** Percentages of distribution of labeled neurons based on their foveal and parafoveal localizations; **(J, K)** Percentages of distribution of labeled neurons located in different layers in foveal V1 **(J)**, and foveal V2 regions **(K)**, shown out of all labeled neurons in V1, V2, and V3; **(L)** An illustration of labeling density, and scaling rates of cluster centers in V1 and V2 relative to the distance of adjacent injection sites in V4. “x” indicates cluster centers in V2, and asterisks indicate cluster centers in V1; **(M)** A simplified illustration of injections and retrograde labeling in the visual cortex.

Referring to the results of the two V1 foveal injection cases above, we planned to inject four retrograde tracers into the foveal and parafoveal V4 regions. The placement of the tracers was guided by the orientation and extension of lunate sulcus (lus) and superior temporal sulcus (sts) (Nakamura et al., 1993; Tanigawa et al., 2010). CTB-555 and CTB-647 were both injected in the vicinity of the vision center, with a distance of about 2.0 mm apart. CTB-488 and BDA were injected about 3.0 mm apart, each targeting alignment parallel to lus (Figures 7A, B). This monkey was used for a separate study involving imaging of the visual cortex. Upon brain removal, we observed cortical deformation attributed to the edge of the installed imaging window, as seen in Figure 7B. Despite this deformation posing challenges to the analysis of V1, we managed to extract data regarding the topographic arrangement.

After histology works of CO staining, Nissl staining, and BDA staining, the sections were visualized by dark-field and bright-field microscopy (Figures 7C–E). According to our estimation, the receptive field of the CTB-555 injection site was slightly upper visual field in the vicinity of the vision center, and that of the CTB-647 was the lower visual field of the foveal visual field. CTB-488 was considered in the foveal field but near the border to the parafoveal field, while BDA was in the parafoveal field. Scattered retrograde labeling of tracers was clearly observed in V1, V2, V3, V4, and other cortical areas (Figures 7F–H). Notably, the clusters of CTB-555 and CTB-647 labeled neurons mixed with each other around the injection sites and scattered to both upper and lower visual quadrants of V2 and V3.

To statistically estimate the possible topographic distribution of labeled cells, we drew lines of presumed foveal visual field, extending half ellipse lines of the foveal V1 border. In our evaluation, ~99.0% of CTB-555 labeled cells and 90.2% of CTB-647 labeled cells were observed in the foveal region, while 85.0% of CTB-488 labeled cells and 96.1% of BDA labeled cells were observed in the parafoveal field (Figure 7I). These results suggest that the closer the injection site is to the vision center of V4, the more labeled neurons are distributed in the foveal visual field, implying that the topographic map is inherited from V1 to extrastriate visual areas.

To quantify differences across layers, we separately counted the number of labeled cells in the foveal V1 and V2 for each tracer and calculated the percentages of them among all labeled cells in V1 and V2 (Figures 7J, K). Although the injection sites of CTB-488 and BDA were slightly distant from the vision center, we still acquired sufficient numbers of labeled cells. According to our analysis, the labeled cells were distributed in all three depths of layers in the foveal V1 and the foveal V2 (Figures 7J, K). There were 14.4%, 19.2%, and 34.0% of CTB-555 labeled cells in supragranular, middle, and infragranular layers of V2, respectively, while they were 11.2%, 6.1%, and 5.1%, respectively, in V1. For CTB-647 labeled cells, there were 7.9% (supra), 12.3% (middle), 19.1% (infra) in V2, while they were 9.8% (supra), 6.0% (middle), and 1.9% (infra) in V1. These results suggest that the feedforward projections to the foveal V4 arise from all layers of the foveal V1 and V2. Furthermore, 22.4% of CTB-555 labeled neurons and 17.7% of CTB-647 labeled neurons were found in the foveal V1 (Figure 7J), while only a small number of labeled cells (0.02% of CTB-555 labeled neurons, and 1.2% of CTB-647 labeled neurons) were found in the parafoveal V1. These results indicated that there were abundant feedforward projections from the foveal V1 to the foveal V4, while there were few feedforward projections from the parafoveal V1 to V4.

To compare the topography of feedforward projections, we calculated the scaling rates between adjacent clusters in V1 and V2, in comparison to V4 injection sites (Figure 7L). As there were apparently two clusters for CTB-555 and CTB-647 labeled neurons each in the lower and upper visual fields, we separately defined their cluster centers segregated by the H.M. line and calculated the averages of the scaling rate. The cluster center locations of CTB-488 and BDA were both in the lower visual field, and they were not in the foveal V2. In our analysis, the scaling rates between CTB-555 and CTB-647, CTB-647 and CTB-488, and CTB-488 and BDA were −0.31, 0.49, and 0.56, respectively, in V2. Although the scaling rate was below 0.0 between CTB-555 and CTB-647 in V2 (−0.22 and −0.42, in the upper and lower visual field, respectively), it was 0.09 in V1. These results suggest that the map is more concentrated in the foveal V4 compared to that in V1 and V2, consistent with the results of V1 injection cases.

Figure 7M shows the simplified map of retinotopy and the summary of results for Case 3. The labeling clusters were observed in V1, V2, V3, V4, and other cortical regions, and the topographical alignment was maintained. Retrograde labeling clusters of CTB-555 and CTB-647 were observed in V1 as well, but those of CTB-488 and BDA were not.

#### Case 4

**Figure 8:**
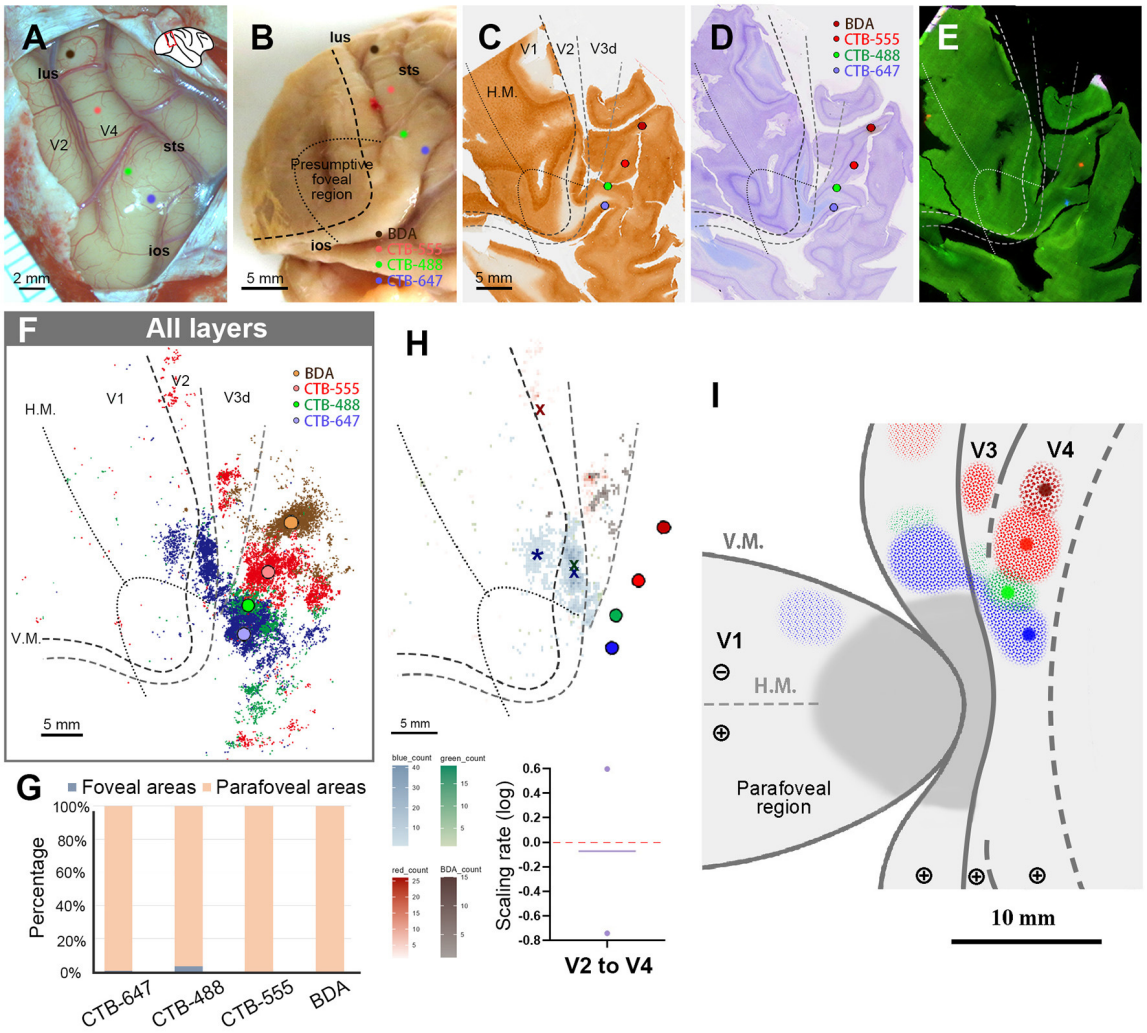
Tracer injections and retrograde labeling in Case 4. **(A)** An image of the foveal V4 at the time of injection with injection sites (blue for CTB-647; green for CTB-488; red for CTB-555; brown for BDA). Right is anterior and upper is dorsal; **(B)** Macaque brain photograph depicting injection sites, presumed V1/V2 border, and foveal region border immediately after extraction; **(C–E)** Tangential sections of flattened V1 stained for CO activity **(C)**, BDA labeling with Nissl substance counterstaining **(D)**, and dark field image of fluorescent labeling **(E)**; **(F)** All labeled cells are plotted in the map of flattened visual cortex. Colored dots represent labeled cells; **(G)** Percentages of distribution of labeled neurons based on their foveal and parafoveal localizations; **(H)** An illustration of labeling density, and scaling rates of cluster centers in V1 and V2 relative to the distance of adjacent injection sites in V4. “x” and an asterisk indicate cluster centers in V1 and V2, respectively; **(I)** A simplified illustration of injections and retrograde labeling in the visual cortex.

In this case, upon opening the dura, we encountered a challenge as the V4 foveal region was in close proximity to the ear bone, making it difficult to target the foveal V4 accurately. We opted to inject CTB-647 near the end of sts, followed by CTB-488, CTB-555, and BDA, each 2.0–3.0 mm apart in parallel to lus (Figures 8A, B). Upon histological examination, we observed that all four tracers were injected in the lower visual quadrant of V4. The injection sites of CTB-647 and CTB-488 were estimated to be near the border of the foveal visual field, while CTB-555 and BDA were placed in the parafoveal V4 (Figures 8C–E). After plotting all neurons, we observed signals spreading into V2, V3, and other cortical areas, as well as V4 near the injection sites (Figure 8F). In V1, there were few labeled cells except CTB-647. In our evaluation, 99.4% of CTB-647 labeled cells, 96.5% of CTB-488 labeled cells, 99.9% of CTB-555 labeled cells, and 100% of BDA labeled cells were located in the parafoveal visual field (Figure 8G). Due to the limited number of labeled cells in the foveal visual field, we did not analyze laminar distribution patterns in this case. Our data implied that there were few direct feedforward projections from the parafoveal V1 to V4.

We then proceeded to compare the topography of feedforward projections from V1 and V2 to V4. In V1, we determined only the center of CTB-647 labeled cells and not of other tracers, as the signals from the other three types of tracers were minimal and too scattered. However, in V2, we were able to pinpoint cluster center locations for CTB-647, CTB-488, and CTB-555 (Figure 8H). In our analysis, the scaling rates between CTB-647 and CTB-488, CTB-488 and CTB-555 were −0.74 and 0.60, respectively, in V2. These two values are largely different, but we realized that their average was nearly zero (−0.07), implying that the topography sizes are comparable between the parafoveal V2 and the parafoveal V4.

Figure 8I illustrates a simplified map of retinotopy and provides a summary of our tracing results in Case 4. In V1, a cluster was observed only for CTB-647. The scaling of clusters was relatively close between V2 and V4 in the parafoveal visual field.

## Discussion

In this study, we investigated the topographic organization of foveal and parafoveal representations in the visual cortex using multiple tracers in each individual animal. We observed direct interconnections between neurons in foveal V1 and foveal V4, while limited direct connectivity existed between V1 and V4 neurons in the parafoveal visual field.

Before conducting the study, we hypothesized four models as shown in Figures 1A–D. However, our results did not align precisely with any of these models. Contrary to the regular concentric organization model (Figure 1A), cortical topography did not exhibit exact continuity along isoeccentricity lines. Additionally, our findings did not support the notion of an ambiguous foveal topography (Figure 1B), as cortical topography was evident even in the foveal visual field. Although some discontinuity in cortical topography was observed, it was not as pronounced as V3 depicted in the discontinuous eccentricity model (Figure 1C). As for the continuous V3 model (Figure 1D), we could not conclusively determine whether V3 is continuous, but at least we did not observe separable clusters from those in V4 in any of the four cases. Regarding layer projection patterns (Figures 1E, F), our data from Case 1 and Case 2 indicated that connectivity from extrastriate areas to V1 follows a regular feedback projection mode, but not intra-areal connectivity mode.

**Figure 9:**
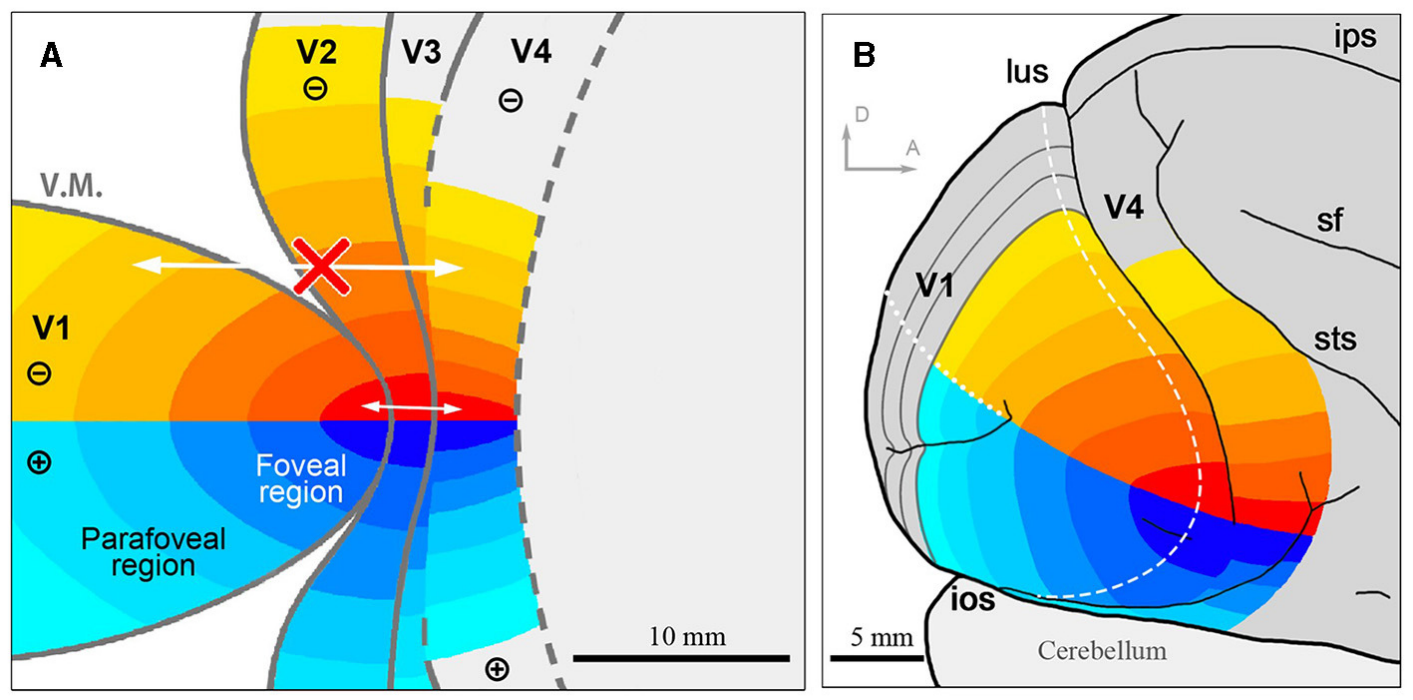
The illustration of the topographic map in the foveal/parafoveal regions of the visual cortex. **(A)** Assumed topographic maps illustrated on the flattened view of the visual cortex. Identical colors of each area indicate shared topography. Arrows indicate that there is intense mutual connectivity in the foveal region, while not outside the foveal region; **(B)** Assumed topographic maps illustrated on the outer surface view of the macaque brain. sf, sylvian fissure; ips, intraparietal sulcus. Scale bars = 10 mm for **(A)**, 5 mm for **(B)**. D and A in **(B)** indicate dorsal and anterior, respectively.

Based on our results, we present our interpretation of topographies in visual areas around the foveal visual field in flattened and unflattened surface views (Figure 9). Note that while we examined topographical connectivity patterns among visual areas, we did not investigate the receptive fields of each region. Since we cannot determine whether V3 is continuous or not at the foveal region, we did not draw clear border of V3. Nonetheless, this study confirms connectivity patterns of the foveal visual field that were previously uncertain.

### Border of V3

Compared to other visual areas, V3 has not been well-characterized. Even the position of its borders remains controversial, lacking a decisive study (Rosa and Tweedale, 2005; Arcaro and Kastner, 2015). Originally proposed as a visual area mirroring V2 in retinotopic organization and extent along the outer border of V2, the evidence came from classical experiments demonstrating anatomical projections from dorsal V1 to both dorsolateral V2 and to a possible V3 immediately rostral to dorsolateral V2 (Zeki, 1969). Subsequently, a modified concept of V3, based on a detailed microelectrode mapping study of extrastriate visual cortex, suggested that V3 was reduced in size and divided into separate dorsal and ventral regions representing the lower and upper visual quadrants, respectively (Gattass et al., 1988). Interareal differences in metabolic activity revealed by CO and myelin staining support the idea of segregated V3 in marmosets and macaques (Gattass et al., 1988; Lyon and Kaas, 2001; Sincich et al., 2003). Some high-resolution fMRI studies targeted the foveal representation of visual areas in humans and suggested that the dorsal and ventral halves of V3 were continuous even in the foveal visual field (Hansen et al., 2007; Schira et al., 2009), although the resolution was not yet high enough to determine area borders in the foveal visual field.

Our current data are not decisive either, but relatively supportive to the discontinuous model, as clusters of retrograde labeling from V1 were observed just outside dorsal parafoveal V2 but apart from clusters in the foveal V4 in Case 1. Perhaps, V3 is continuous within the foveal visual field as well, but even if so, the foveal V3 region would be very thin.

Moreover, throughout the four cases here, V1 showed direct connectivity with V4 in the foveal visual field, but much fewer in the parafoveal visual field. This is likely because the parafoveal V1 is separated from the parafoveal V4 by the relatively thick V3, as well as V2, while the foveal V1 is only separated from the foveal V4 by V2. Direct connectivity between the foveal V1 and the foveal V4 was previously revealed by neuronal tracing studies (Zeki, 1978; Nakamura et al., 1993; Ungerleider et al., 2008), but the current study has more systematically shown a dramatic shift of connectivity across the foveal and parafoveal visual fields regarding direct mutual connectivity between V1 and V4. Note that this lack of direct connectivity does not imply that parafoveal V4 receives no visual information from V1. Our study solely examined direct connectivity and did not explore indirect connectivity pathways. It is likely that parafoveal V1 sends inputs to parafoveal V2 and V3, which in turn relay information to parafoveal V4. Similarly, foveal V1 is expected to project to foveal V2 and V4. This delineation suggests that feedforward and feedback connectivity extend up to two hierarchical steps, but not beyond.

### Supra-areal eccentricity organization model in the foveal visual field

The “supra-areal eccentricity organization model” illustrates large-scale coherence, emphasizing local continuity across individual visual field maps (Buckner and Yeo, 2014). This idea initially emerged from electrophysiological mapping studies across occipital cortical areas in monkeys (Rosa, 2002) and was further emphasized with large-scale imaging studies (Kolster et al., 2014). In particular, the eccentricity organization of the foveal visual field from V1 to V4 is contiguous, forming an elongated strip that disregards areal boundaries (Arcaro and Kastner, 2015).

However, our current study raises questions about this idea. We examined layer differences because we wondered whether the layer projection patterns among visual areas in the foveal visual field follow inter-areal patterns or intra-areal patterns. Our data suggest that feedback projections from the foveal V4 to the foveal V1, and from the foveal V2 to the foveal V1, predominantly arise from infragranular layers. While distinguishing between feedforward projection and intra-areal connectivity patterns proved challenging, there was no particular question to believe that projections from the foveal V1 to the foveal V4 in our data followed regular inter-areal feedforward projection mode. This implicates that projection patterns among visual areas are more in line with the inter-areal mode, even in the foveal visual fields.

Furthermore, our data demonstrate that isoeccentricity lines are not continuous from V1 to V4, with the foveal V3 and V4 being more concentrated than the foveal V1 and V2 (Figure 9). The concentrated retinotopy of V4 is evident in images from previous BOLD visuotopic mapping studies as well (Fize et al., 2003; Arcaro et al., 2011; Kolster et al., 2014; Wang et al., 2015), although these authors did not emphasize it. While foveal visual fields in V1–V4 are firmly interconnected, they still appear as independent cortical areas, cautioning against oversimplifying brain organization.

### Considerations regarding development and evolution

In the context of primate visual area development, the “two-seeds theory” has been proposed (Rosa, 2002). According to this theory, two primary visual areas with the first-order representation of retinotopy, V1 and MT, are specified early in development, either through gradual distributions of cell surface chemical cues (O'Leary and Nakagawa, 2002) or by the spatio-temporal patterning of afferent projections (Molnar and Blakemore, 1995). Once the V1 and MT maps are defined, visuotopic maps in adjacent areas begin to self-organize around these anchors. Two rules guide this process: (1) the receptive fields of neurons in adjacent columns must overlap; and (2) the gradient of representation does not revert within a given area. Throughout postnatal development, activity-dependent mechanisms fine-tune the maps.

In line with this theory, the maturation of neurochemical components occurs first in V1 and MT, followed by extrastriate visual areas, mirroring retinotopic maps (Bourne and Rosa, 2006; Turner et al., 2020). Considering this developmental progress, it might be reasonable to assume that V1, V2, V3, and V4 all share a retinotopic eccentricity map. However, there is no evidence supporting the idea that the topography of extrastriate areas is solely based on V1 and MT, although it is plausible that the connectivity between V1 (MT) and extrastriate areas plays a role in refining their topography together. A more recent study in rodents proposed a different theory. A detailed early postnatal cortical mapping study using multiphoton calcium imaging in mice demonstrated that retinotopic maps develop simultaneously in V1 and multiple higher visual areas, followed by their interconnection (Murakami et al., 2022). During the early postnatal period, V1 and higher visual areas develop through afferent inputs from the lateral geniculate nucleus (LGN) and the lateral posterior nucleus (LPN, the counterpart of the primate pulvinar), respectively. This model seems reasonable when considering the time needed to develop the entire visual system. If hierarchical development from one area to another were the case, it would take an extended period to complete the development of all cortical areas.

While one might argue that visual systems differ significantly between rodents and primates, recent studies indicate that even rodent visual systems share more homologous characteristics with primate visual systems than previously estimated. For instance, there is a distinction between dorsal and ventral visual pathways both anatomically and physiologically in rodents (Murakami et al., 2017). Ocular dominance columns (ODCs), once thought to be unique to primates and carnivores, have been discovered in rats, and their developmental events appear similar to those in primate/carnivore ODCs (Zhou et al., 2023). Thus, rodent brains can be considered prototypes of primate brains, despite lacking a prominent fovea. V2, V3, V4, and MT have all been demonstrated topographically organized, intense mutual connectivity with the pulvinar (Kaas and Baldwin, 2019; Liu et al., 2021). It is possible that V3 and V4 develop topographic maps independently of V1, although they are delayed compared to V1 and MT, via topographic cues from the pulvinar. The foveal region likely exhibits strong activity, leading to closely located foveal visual fields in each area.

## Data availability statement

The original contributions presented in the study are included in the article/supplementary material, further inquiries can be directed to the corresponding author.

## Ethics statement

The animal study was approved by the Institutional Animal Care and Use Committee of Zhejiang University. The study was conducted in accordance with the local legislation and institutional requirements.

## Author contributions

HL: Data curation, Formal analysis, Investigation, Methodology, Project administration, Validation, Writing – original draft, Writing – review & editing. DH: Data curation, Formal analysis, Investigation, Methodology, Writing – original draft. HT: Data curation, Formal analysis, Funding acquisition, Investigation, Methodology, Supervision, Validation, Writing – original draft. TT: Conceptualization, Data curation, Formal analysis, Funding acquisition, Investigation, Methodology, Project administration, Resources, Supervision, Validation, Visualization, Writing – original draft, Writing – review & editing.
